# Performance evaluation of four antibiotics using the BD Phoenix™ NMIC-413 antimicrobial susceptibility testing panel for carbapenem-resistant *Enterobacteriaceae* and carbapenem-resistant *Pseudomonas aeruginosa*

**DOI:** 10.3389/fmicb.2025.1593674

**Published:** 2025-07-04

**Authors:** Yajuan Guan, Zhenli Song, Yanshan Chen, Jiayu Feng, Yizhi Fan, Yongyu Rui

**Affiliations:** ^1^Department of Laboratory Medicine, Guangdong Provincial Key Laboratory of Precision Medical Diagnostics, Guangdong Engineering and Technology Research Center for Rapid Diagnostic Biosensors, Guangdong Provincial Key Laboratory of Single-cell and Extracellular Vesicles, Nanfang Hospital, Southern Medical University, Guangzhou, China; ^2^Guangdong Provincial Clinical Research Center for Laboratory Medicine, Guangzhou, China; ^3^Zhuhai Health School, Zhuhai, China; ^4^Fuwai Central China Cardiovascular Hospital, Zhenzhou, China

**Keywords:** BD Phoenix™ NMIC-413 panel, CRE, CRPA, antimicrobial susceptibility, evaluation

## Abstract

**Background:**

The spread of antimicrobial resistance (AMR) poses significant threats to human health. In 2024, the World Health Organization (WHO) classified carbapenem-resistant *Enterobacteriaceae* (CRE) as a critical-priority pathogen and carbapenem-resistant *Pseudomonas aeruginosa* (CRPA) as a high-priority pathogen. This study aimed to evaluate the performance of meropenem (MEM), imipenem (IPM), cefepime (FEP), and cefoperazone/sulbactam (SCF) using the BD Phoenix™ NMIC-413 antimicrobial susceptibility testing (AST) panel (NMIC-413 panel) for CRE and CRPA at Nanfang Hospital, China.

**Methods:**

A total of 314 archived Gram-negative clinical isolates were tested, including 219 *Enterobacteriaceae* isolates (150 CRE) and 95 *P. aeruginosa* isolates (56 CRPA). The NMIC-413 panel and the disk diffusion method were employed for AST of MEM, IPM, FEP, and SCF. Broth microdilution (BMD) was used as the reference method. Categorical agreement (CA), essential agreement (EA), very major errors (VME), major errors (ME), and minor errors (MIE) were calculated. The acceptable standards were as follows: CA and EA > 90%, ME < 3%, and VME < 1.5%.

**Results:**

For CRE, the NMIC-413 panel met the acceptable standards and demonstrated higher CA values than the disk diffusion method for all four antibiotics (99.3, 96.6, 98.0, and 98.7% vs. 98.7, 96.0, 96.0, and 97.3%, respectively). For CRPA, the NMIC-413 panel also met the acceptable standards and showed superior CA values for MEM and FEP compared to the disk diffusion method (98.2 and 96.4% vs. 96.4 and 92.9%, respectively), while CA values for IPM and SCF were similar between the two methods (98.2 and 92.9% vs. 98.2 and 92.9%, respectively).

**Conclusion:**

The NMIC-413 panel demonstrated Clinical Laboratory Standards Institute (CLSI)-compliant performance for all four tested antibiotics against CRE and CRPA, exhibiting superior reliability compared to the conventional disk diffusion method. Future studies should focus on establishing standardized breakpoints for SCF, expanding the detection spectrum for rare bacterial species, and conducting multicenter validation to assess regional variations. We recommend the NMIC-413 panel for AST of CRE and CRPA isolates as a practical alternative to the BMD method.

## 1 Introduction

Bacterial infections pose a significant challenge to global public health. A recent study published in *The Lancet* (Ikuta et al., 2022) revealed that in 2019, approximately 7.7 million deaths worldwide were associated with 33 common bacterial pathogens, with over half of these deaths attributed to five major species: *Staphylococcus aureus*, *Escherichia coli*, *Streptococcus pneumoniae*, *Klebsiella pneumoniae*, and *Pseudomonas aeruginosa*. In China, between 2005 and 2022, *Enterobacteriaceae* and non-fermentative bacilli were the predominant isolates found in bacterial infections, with *Escherichia coli*, *Klebsiella pneumoniae*, and *Pseudomonas aeruginosa* (*P. aeruginosa*) ranking among the top five pathogens (Qin et al., 2024).

Antimicrobial resistance (AMR) related to bacterial infections has also garnered global attention. Naghavi et al. (2024) reported that in 2021, an estimated 4.71 million deaths were linked to bacterial AMR. Among Gram-negative bacteria, resistance to carbapenem antibiotics was responsible for more deaths than any other class of antibiotics, with the number of associated deaths rising from 619,000 in 1990 to 1.03 million in 2021. Carbapenem-resistant Gram-negative bacteria (CRGNB) primarily include carbapenem-resistant *Enterobacteriaceae* (CRE) and carbapenem-resistant *P. aeruginosa* (CRPA). CRE refers to a group of *Enterobacteriaceae* that develop resistance to carbapenem antibiotics by producing carbapenemases. Since the discovery of carbapenem-resistant *Klebsiella pneumoniae* (CRKP) in the United States in 2001 (Yigit et al., 2001), CRE has rapidly spread worldwide (Potter et al., 2016). According to the 2021 European Antimicrobial Resistance Surveillance Network report, only 2 of 44 countries had carbapenem resistance rates in *P. aeruginosa* below 5%, whereas 6 countries reported rates of50% or higher (Report E, 2023). Due to their clinical significance, the WHO classified CRE as a critical-priority pathogen and CRPA as a high-priority pathogen in 2024 (WHO, 2024). CRE and CRPA pose major challenges in controlling nosocomial infections owing to their high resistance to various antibiotics. These pathogens not only limit available treatment options but also lead to increased medical costs and a higher risk of patient mortality (Ernst et al., 2020; Tenover et al., 2022).

The *Infectious Diseases Society of America (IDSA)* 2022 guidelines on the treatment of CRE and difficult-to-treat resistance (DTR) *P. aeruginosa* recommend the following (Tamma et al., 2022): For CRE infections, combination therapy with carbapenems could be selected based on AST, and carbapenems could be used in combination with enzyme inhibitors to delay resistance development. For DTR *P. aeruginosa*, when isolates demonstrate susceptibility to traditional non-carbapenem *β*-lactams (e.g., cefepime), these agents are preferred over carbapenems. Rhodes et al. (2017) advocated for immediate (<1 h) intravenous broad-spectrum empirical therapy—including carbapenems, penicillin/*β*-lactamase inhibitors, or advanced cephalosporins—prior to AST result availability in cases of sepsis or septic shock. In 2023, a multidisciplinary panel of 31 Chinese clinical experts established guidelines for the diagnosis and treatment of infections caused by carbapenem-resistant Gram-negative bacilli (CRGNB) (Zeng et al., 2023). They recommended carbapenem-based combination regimens or sulbactam-containing regimens for CRGNB infections. The WHO AWaRe (Access, Watch, Reserve) antibiotic manual classified carbapenems and certain third- and fourth-generation cephalosporins as “Watch” or “Reserve” group antibiotics for various infections (Moja et al., 2024). Based on AST results, these agents were listed as first-line, second-line, or “last-resort” antibiotics for specific infectious syndromes. However, due to their higher potential for driving antimicrobial resistance, they were prioritized as targets for stewardship programs and surveillance.

In recent years, various methods have been developed to detect bacterial antimicrobial susceptibility (Datar et al., 2022), including disk diffusion, the T^2^Bacteria panel (Nguyen et al., 2019), and Cas9-coupled nanopore sequencing-enhanced mNGS technology (Serpa et al., 2022). However, rapid and accurate determination of the minimum inhibitory concentration (MIC) of antibiotics remains crucial for guiding precise clinical treatment. The BD Phoenix™ NMIC-413 AST panel (hereinafter referred to as the NMIC-413 panel), launched by BD in 2018, was designed for MIC detection in Gram-negative bacteria, encompassing critical clinical antibiotics such as carbapenems and major cephalosporins. However, its performance in detecting susceptibility to carbapenems and key cephalosporins has not been thoroughly evaluated. This study assessed the performance of the NMIC-413 panel for four antibiotics—meropenem (MEM), imipenem (IPM), cefepime (FEP), and cefoperazone/sulbactam (SCF)—against *Enterobacteriaceae* (including CRE) and *P. aeruginosa* (including CRPA) isolates collected from Nanfang hospital in Southern China. The broth microdilution (BMD) method, following the CLSI M52 (Clinical and Laboratory Standards Institute, 2015) guidelines, was used as the reference method to validate the susceptibility results of these four antibiotics. In addition, disk diffusion (Kronvall et al., 2011), a simple and cost-effective technique widely adopted in clinical laboratories worldwide since the 1940s, was included as a comparative method in this evaluation.

## 2 Materials and methods

### 2.1 Isolates

A total of 314 bacterial strains, including 219 *Enterobacteriaceae* and 95 *P. aeruginosa* strains, were selected as research data. These strains were isolated from clinical specimens collected between 2018 and 2023 at the Microbiology Department of Laboratory Medicine, Nanfang Hospital, Southern Medical University, Guangdong Province, China. Among the 219 *Enterobacteriaceae* strains, *Klebsiella pneumoniae* (154 strains) accounted for the highest proportion, followed by *Escherichia coli* (37 strains), *Serratia marcescens* (13 strains), *Enterobacter cloacae* (11 strains), *Klebsiella aerogenes* (two strains), *Citrobacter koseri* (one strain), and *Enterobacter hodginsii* (one strain). The strains were primarily isolated from sputum (47%), urine (12%), blood (9%), and alveolar lavage fluid (8%). They originated from patients in the intensive care unit (ICU) (14%), respiratory department (13%), neurology department (11%), neurosurgery department (10%), hematology department (6%), and general surgery ward (6%) (Supplementary Table S1).

Among the isolates, there were 150 CRE strains, including 132 *Klebsiella pneumoniae* strains, 14 *Escherichia coli* strains, two *Enterobacter cloacae* strains, one *Serratia marcescens* strain, and one *Enterobacter hodginsii* strain. The isolates were identified using MALDI-TOF MS (Vitek MS, BioMérieux, France). All isolates were preserved in cryovials containing 20% (v/v) sterile glycerol and stored at −80°C until subculturing on blood agar plates (Autobio, Henan, China).

### 2.2 Broth microdilution (BMD) method

The BMD method was performed for MEM, IPM, FEP, and SCF in accordance with the CLSI M07 and M100 guidelines (Clinical and Laboratory Standards Institute, 2021; Clinical and Laboratory Standards Institute, 2018), serving as the gold standard in this study. A 0.5 McFarland standard suspension was prepared and inoculated into the corresponding BMD antimicrobial susceptibility panels. Cation-adjusted Mueller-Hinton (MH) broth containing different concentrations of the antibiotics was prepared for the BMD AST panel. MICs were recorded after incubation at 35°C for 16–20 h. The breakpoints are shown in Table 1.

**Table 1:** Breakpoints used in this study.

	*Enterobacteriaceae*						*P. aeruginosa*					
Antibiotics	MIC breakpoints^a^ (ug/mL)			Disk diffusion breakpoints(mm)			MIC breakpoints (ug/mL)			Disk diffusion breakpoints (mm)		
	S^b^	I^c^	R^d^	S	I	R	S	I	R	S	I	R
MEM	≤ 1	2	≥ 4	≥ 23	20–22	≤ 19	≤ 2	4	≥ 8	≥ 19	16–18	≤ 15
IPM	≤ 1	2	≥ 4	≥ 23	20–22	≤ 19	≤ 2	4	≥ 8	≥ 19	16–18	≤ 15
FEP	≤ 2	4–8	≥ 16	≥ 25	19–24	≤ 18	≤ 8	16	≥ 32	≥ 18	15–17	≤ 14
SCF	≤ 16	32	≥ 64	≥ 21	16–20	≤ 15	≤ 16	32	≥ 64	≥ 21	16–20	≤ 15

a MIC breakpoints: The breakpoint for drug susceptibility determination in the BMD method and BD drug susceptibility plate. As the breakpoint interpretation standards for the BMD method and the BD drug susceptibility plate are the same, they are merged together for the sake of conciseness and effectiveness.

b Susceptible.

c Intermediate.

d Resistant.

### 2.3 BD Phoenix system

The 0.5 McFarland standard bacterial suspension was prepared. Subsequently, 25 μL of the bacterial suspension and 50 μL of the Phoenix indicator were sequentially added to the matched Phoenix AST broth. After thorough mixing, the broth mixture was dispensed into the NMIC-413 panels. The minimum inhibitory concentrations (MICs) of MEM, IPM, FEP, and SCF were determined according to the manufacturer’s instructions. The breakpoints are shown in Table 1.

### 2.4 Disk diffusion method

Experiments were performed using the disk diffusion method with MEM (10 μg), IPM (10 μg), FEP (30 μg), and SCF (75/30 μg) disks (Oxoid, Basingstoke, UK) in accordance with the CLSI M02 and M100 guidelines (Clinical and Laboratory Standards Institute, 2021; Clinical and Laboratory Standards Institute, 2024). Sterile cotton swabs were dipped into the bacterial solution and evenly smeared on the agar surface. Sterile tweezers were used to place medicated paper onto the agar surface. After incubation at 35°C for 16–18 h, the inhibition zone was measured with a vernier caliper, and the breakpoints are shown in Table 1.

### 2.5 Quality control (QC)

Daily QC testing was performed according to the CLSI guidelines (Clinical and Laboratory Standards Institute, 2015; Clinical and Laboratory Standards Institute, 2021; Clinical and Laboratory Standards Institute, 2018; Clinical and Laboratory Standards Institute, 2024), using ATCC 25922 *E. coli* and ATCC 27853 *P. aeruginosa* strains. Results were considered invalid if QC values were outside the specified range.

### 2.6 Interpretation of the antimicrobial susceptibility results

Table 1 was created according to the CLSI guidelines (Clinical and Laboratory Standards Institute, 2015; Clinical and Laboratory Standards Institute, 2021; Clinical and Laboratory Standards Institute, 2024) to present the breakpoints for the three methods (MIC breakpoints for the BMD, NMIC-413 panel, and disk diffusion methods) to compare the antimicrobial susceptibility results of the NMIC-413 panel and disk diffusion method with those of the BMD method. Current CLSI/EUCAST guidelines lack established AST breakpoints for SCF against *Enterobacteriaceae* and *P. aeruginosa*. Some scholars (Barry and Jones, 1988; Jean et al., 2017; Jones et al., 1987; Jones et al., 2014) have recommended adopting the cefoperazone susceptibility breakpoints from CLSI. Barry and Jones (1988) evaluated the performance of cefoperazone disks containing 15 μg or 30 μg of sulbactam and SCF broth microdilution tests for AST of *Escherichia coli*, *P. aeruginosa*, *Staphylococcus aureus*, and other bacteria. The study suggested that the breakpoints recommended for cefoperazone testing could also be applied to SCF. Jean et al. (2017) proposed the following SCF breakpoints against *Enterobacteriaceae* and *P. aeruginosa*: for disk diffusion (75/30 μg), inhibition zone diameter ≥ 21 mm (susceptible), 16-20 mm (intermediate), and ≤ 15 mm (resistant); for BMD, MICs ≤ 16 μg/mL (susceptible), 32 μg/mL (intermediate), and ≥ 64 μg/mL (resistant). These breakpoints are consistent with CLSI’s cefoperazone breakpoints against *Enterobacteriaceae* (Clinical and Laboratory Standards Institute, 2021) and *P. aeruginosa* (Clinical and Laboratory Standards Institute, 2010). The MIC breakpoints for the four antibiotics against *Enterobacteriaceae* and *P. aeruginosa* are shown in Table 1.

### 2.7 Data analysis

Origin 2022 (OriginLab, USA) and SPSS 25.0 (IBM, USA) were used to process the experimental data. Five evaluation indicators were calculated: categorical agreement (CA), essential agreement (EA), very major errors (VME), major errors (ME), and minor errors (MIE). CA and EA values > 90%, ME values < 3%, and VME values < 1.5% are considered acceptable according to the CLSI guidelines (Clinical and Laboratory Standards Institute, 2015; Humphries et al., 2018). The results were classified as CA when the two MICs determined by the evaluated method and the reference method were sensitive, intermediate, or resistant. The results were classified as EA when the difference between the two MICs determined by the NMIC-413 panel and the BMD method was less than one dilution. The results were classified as ME when the MIC determined by the BMD method was sensitive and the MIC determined by the NMIC-413 panel or the disk diffusion method was resistant. The results were classified as MIE when one MIC was intermediate and the other was either sensitive or resistant. The results were classified as VME when the MIC determined by the BMD method was resistant and the MIC determined by the NMIC-413 panel or disk diffusion method was sensitive.

In this study, we calculated linear regression curves of log-transformed MIC values between the BMD method and the NMIC-413 panel, as well as between the BMD method and the disk diffusion method, to evaluate and compare their correlations. The closer the R^2^ value was to 1, the better the regression fitting effect. A *p*-value of <0.05 indicated a statistically significant relationship between the independent variable and the dependent variable. In addition, we assessed the inter-rater reliability between the BMD method and NMIC-413 and between the BMD method and the disk diffusion method using Kappa statistics. A Kappa value closer to 1 suggested stronger agreement in the classification results. In addition, a *p*-value <0.05 was considered statistically significant. Finally, we presented the specific distributions of MIC values between the BMD method and the NMIC-413 panel, as well as between the BMD method and the disk diffusion method. Since the NMIC-413 panel and BMD shared consistent MIC units, their dilution gradient consistency and differences could be evaluated. All abbreviations were defined upon first use and used consistently throughout the study.

## 3 Results

### 3.1 Bacterial susceptibility test results

The test results for all strains are presented in Supplementary Table S1. The performance of the NMIC-413 panel and the disk diffusion method was evaluated against the BMD method for MEM, IPM, FEP, and SCF by calculating CA, EA, VME, ME, and MIE values. To provide a detailed overview of the bacterial susceptibility testing performance of the NMIC-413 panel, the results for all bacteria, *Enterobacteriaceae*, carbapenem-sensitive *Enterobacteriaceae* (CSE), CRE, *Klebsiella pneumoniae*, carbapenem-sensitive *Klebsiella pneumoniae* (CSKP), CRKP, carbapenem-sensitive *P. aeruginosa* (CSPA), and CRPA, are shown in Supplementary Tables S2–S5. For *Enterobacteriaceae* and *P. aeruginosa*, the AST results using the NMIC-413 panel for MEM (*Enterobacteriaceae*: CA 99.5%, EA 98.2%, ME 0.5%, and VME 0%; *P. aeruginosa*: CA 95.8%, EA 92.6%, ME 0%, and VME 0%) (Supplementary Table S2), IPM (*Enterobacteriaceae*: CA 96.3%, EA 93.6%, ME 0.9%, and VME 0.9%; *P. aeruginosa*: CA 97.9%, EA 95.8%, ME 0%, and VME 0%) (Supplementary Table S3), FEP (*Enterobacteriaceae*: CA 97.7%, EA 97.7%, ME 0%, and VME 0%; *P. aeruginosa*: CA 96.8%, EA 91.6%, ME 1.1%, and VME 0%) (Supplementary Table S4), and SCF (*Enterobacteriaceae*: CA 98.2%, EA 94.5%, ME 1.4%, and VME 0%; *P. aeruginosa*: CA 93.7%, EA 92.6%, ME 2.1%, and VME 0%) (Supplementary Table S5) fully complied with the acceptable standards.

**Figure 1:**
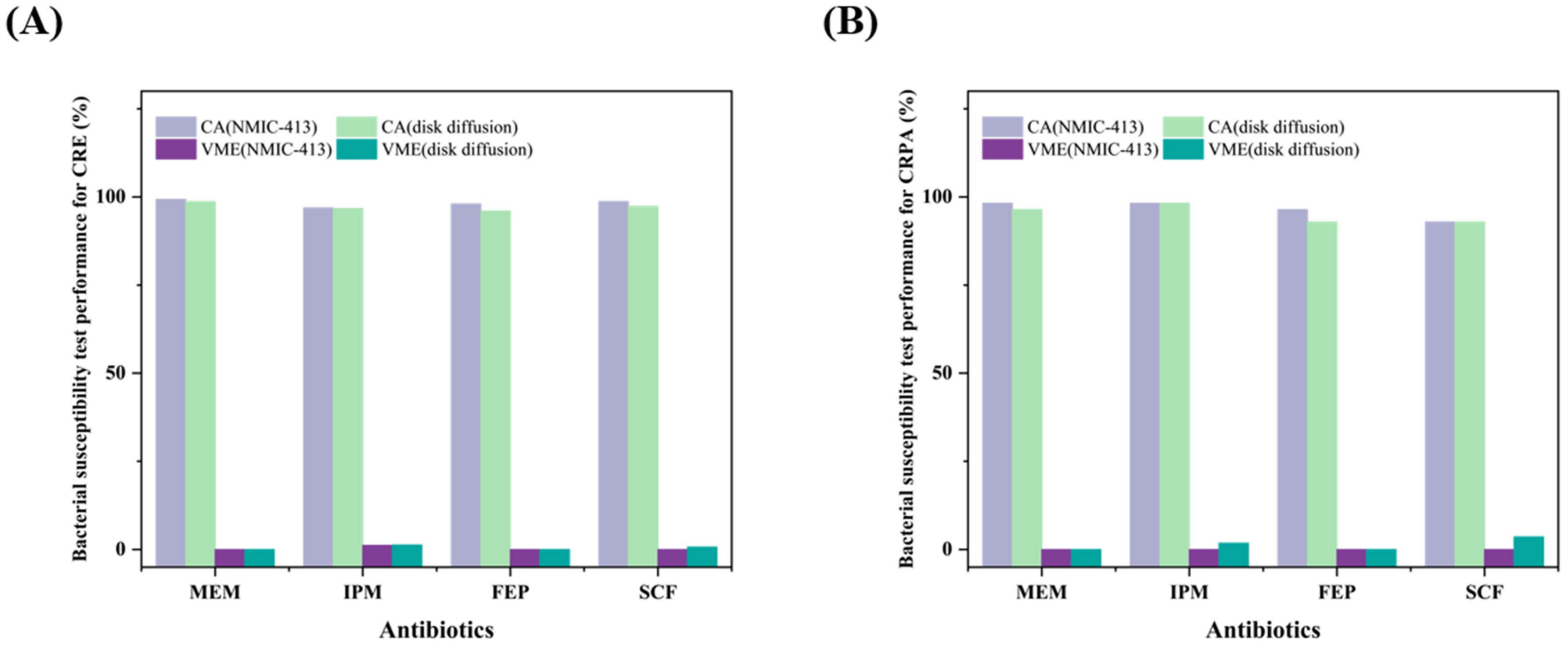
Comparison of CA and VME values between the NMIC-413 panel and the disk diffusion method for carbapenem-resistant *Enterobacteriaceae* (CRE) **(A)** and carbapenem-resistant *Pseudomonas aeruginosa* (CRPA) **(B)**.

Tables 2, 3, along with Figure 1, summarize the statistical results for CRE and CRPA. Figure 1 presents a bar chart comparing the CA and VME values of the four antibiotics listed in Tables 2, 3. For CRE (Table 2), the CA, EA, ME, and VME values of MEM (CA 99.3%, EA 98.7%, ME 0.7%, and VME 0%), IPM (CA 96.6%, EA 96.0%, ME 1.3%, and VME 1.3%), FEP (CA 98.0%, EA 97.3%, ME 0%, and VME 0%), and SCF (CA 98.7%, EA 97.3%, ME 1.3%, and VME 0%), as determined by the NMIC-413 panel, all met the acceptable standards. In addition, the CA values of the four antibiotics determined by the NMIC-413 panel were all higher than those determined by the disk diffusion method (NMIC-413: 99.3, 96.6, 98.0, and 98.7% vs. disk diffusion: 98.7, 96.0, 96.0, and 97.3%, respectively). For CRPA (Table 3), the NMIC-413 panel met the acceptable standards for all antibiotics tested: MEM (CA 98.2%, EA 96.4%, ME 0%, VME 0%), IPM (CA 98.2%, EA 98.2%, ME 0%, VME 0%), FEP (CA 96.4%, EA 92.9%, ME 1.8%, VME 0%), and SCF (CA 92.9%, EA 94.6%, ME 1.8%, VME 0%). For MEM and FEP, the categorical agreement values determined by the NMIC-413 panel were higher than those obtained using the disk diffusion method (98.2% vs. 96.4% for MEM; 96.4% vs. 92.9% for FEP). Regarding IPM and SCF, the values were identical for both methods.

**Table 2:** Performance of bacterial susceptibility testing for carbapenem-resistant *Enterobacteriaceae* (CRE).

	NMIC-413 panel						Disk diffusion method				
Antibiotics	Total no. evaluable	CA^a^ (%)	EA^b^ (%)	VME^c^ (%)	ME^d^ (%)	MIE^e^ (%)	Total no. evaluable	CA (%)	VME (%)	ME (%)	MIE (%)
MEM	150	99.3	98.7	0	0.7	0	150	98.7	0	0	1.3
IPM	149	96.6	96.0	1.3	1.3	0.7	150	96.0	1.3	0.7	2.0
FEP	150	98.0	97.3	0	0	2.0	150	96.0	0	0	4.0
SCF	150	98.7	97.3	0	1.3	0	150	97.3	0.7	1.3	0.7

a Categorical agreement.

b Essential agreement.

c Very major errors.

d Major errors.

e Minor errors.

**Table 3:** Performance of bacterial susceptibility testing for carbapenem-resistant *P. aeruginosa* (CRPA).

	NMIC-413 panel						Disk diffusion method				
Antibiotics	Total no. evaluable	CA (%)	EA (%)	VME (%)	ME (%)	MIE (%)	Total no. evaluable	CA (%)	VME (%)	ME (%)	MIE (%)
MEM	56	98.2	96.4	0	0	1.8	56	96.4	0	0	3.6
IPM	56	98.2	98.2	0	0	1.8	56	98.2	1.8	0	0
FEP	56	96.4	92.9	0	1.8	1.8	56	92.9	0	0	7.1
SCF	56	92.9	94.6	0	1.8	5.4	56	92.9	3.6	0	3.6

### 3.2 Statistical analysis

**Figure 2:**
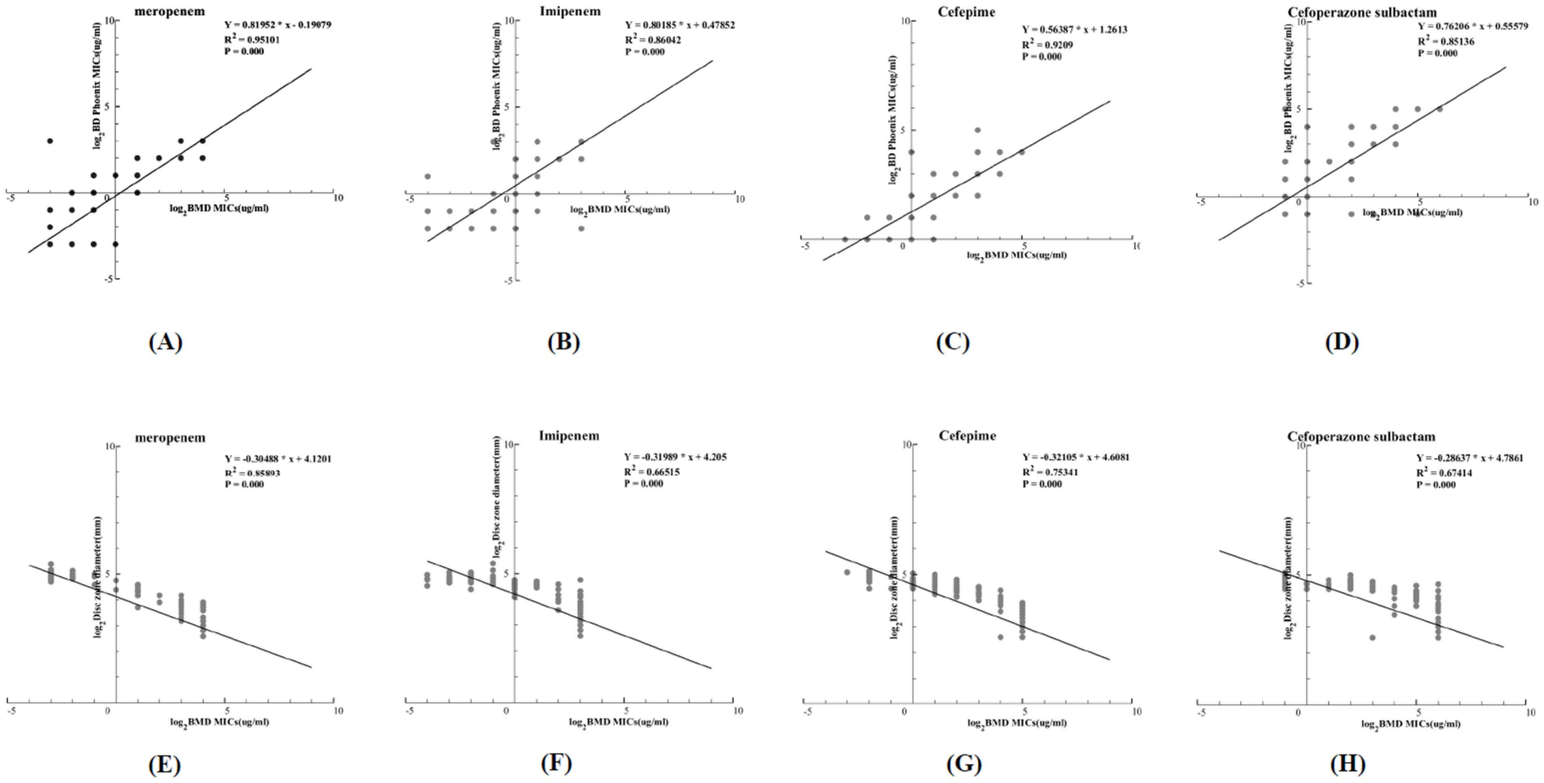
**(A–D)**: Regression curves comparing the MICs obtained by the BD Phoenix™ NMIC-413 panel with those from the BMD method; **(E–H)**: Regression curves comparing the disk zone diameter of the disk diffusion method with MICs from the BMD method.

Linear regression curves comparing the detection results of the BMD method and the NMIC-413 panel are shown in Figures 2A–D. The *p*-values for MEM, IPM, FEP, and SCF were all <0.001, and the R^2^ values were 0.95101, 0.86042, 0.9209, and 0.85136, respectively, indicating that the NMIC-413 panel showed a better regression fitting effect in detecting MEM. Linear regression curves comparing the detection results of the BMD and disk diffusion methods are shown in Figure 2E–H. The *p*-values for MEM, IPM, FEP, and SCF were all <0.001, and the R^2^ values were 0.85893, 0.66515, 0.75341, and 0.67414, respectively—all lower than those obtained with the NMIC-413 panel. This demonstrated that the NMIC-413 panel provided better regression fitting for all four antibiotics compared to the disk diffusion method.

**Figure 3:**
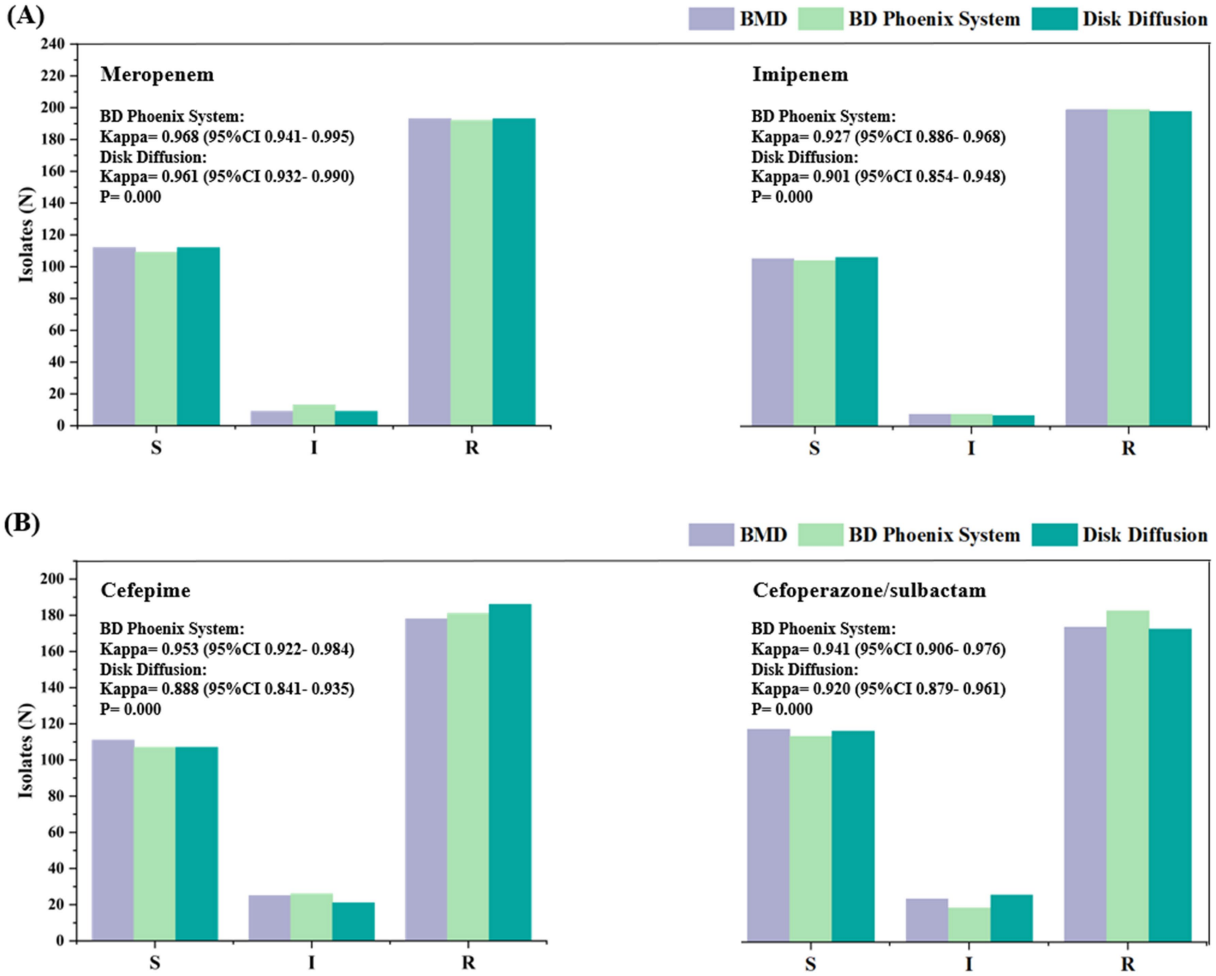
Susceptibility of the 314 isolates to meropenem **(A)** (left), imipenem **(A)** (right), cefepime **(B)** (left), and cefoperazone/sulbactam **(B)** (right). The labels in this figure display the Kappa values comparing the antimicrobial susceptibility categorization results between the BD Phoenix NMIC-413 system/disk diffusion method and the reference method (BMD).

The susceptibility profiles and kappa statistics for the four antibiotics tested using the different methods (between the BMD method and the NMIC-413 panel and between the BMD method and the disk diffusion method) are shown in Figure 3. According to the BMD method, the number of resistant strains to MEM, IPM, FEP, and SCF was 193, 200, 178, and 173, respectively. All comparisons demonstrated highly significant concordance (*p* < 0.001). The NMIC-413 panel yielded kappa values of 0.968 for MEM, 0.927 for IPM, 0.953 for FEP, and 0.941 for SCF, whereas the disk diffusion method showed corresponding values of 0.961 (MEM), 0.901 (IPM), 0.888 (FEP), and 0.920 (SCF). All results exhibited excellent agreement (Kappa > 0.8).

The Kappa values for the NMIC-413 panel were consistently higher than those for the disk diffusion method, indicating stronger concordance between the NMIC-413 panel and the BMD method for AST. Notably, the NMIC-413 panel exhibited a significant advantage in detecting FEP (ΔKappa = +0.065), likely attributable to its MIC-based quantitative nature, which reduces the subjective variability inherent in inhibition zone interpretation with the disk diffusion method. Furthermore, IPM showed slightly lower Kappa values (NMIC-413: 0.927 vs. disk diffusion: 0.901), suggesting that the classification of isolates near the breakpoint requires cautious validation. These findings support the reliability of automated AST systems in clinical laboratories, particularly for MEM, FEP, and SCF, while highlighting the need for improved standardization in conventional methods.

**Figure 4:**
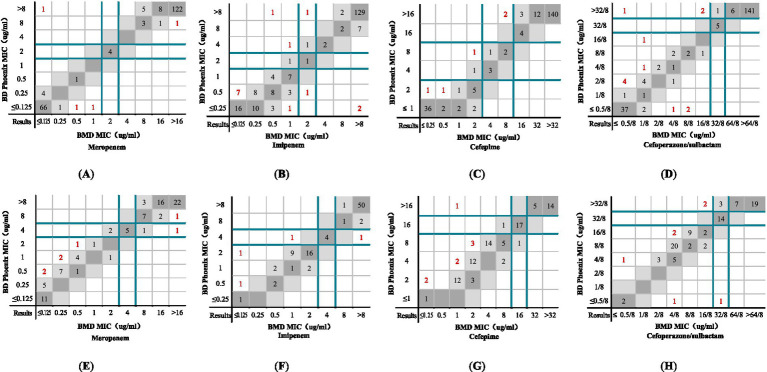
MICs determined by the NMIC-413 panel versus the BMD method: **(A)** MEM, **(B)** IPM, **(C)** FEP, and **(D)** SCF show the comparison results of *Enterobacteriaceae* determined by the NMIC-413 panel and BMD method; **(E)** MEM, **(F)** IPM, **(G)** FEP, and **(H)** SCF show the comparison results of *P. aeruginosa* determined by the NMIC-413 panel and BMD method. Dark grey indicates identical MIC values, while light grey represents a twofold difference between the MICs obtained by the NMIC-413 panel and the BMD method. Dark green lines show the clinical breakpoints for each antibiotic, and red markers indicate outliers where the NMIC-413 results differed by >2-fold dilution compared to the BMD method.

The distribution of MICs determined by the BMD method and the NMIC-413 panel for *Enterobacteriaceae* and *P. aeruginosa* are shown in Figures 4A–H. The majority of the differences in MIC values between the NMIC-413 panel and the BMD method were ≤2-fold dilution. Compared to MEM, IPM, and FEP, the MIC distribution for SCF was more uniform. Figure 4 highlights in red the outliers where the NMIC-413 panel showed >2-fold dilution discrepancies compared to the BMD method. Among the *Enterobacteriaceae* isolates, the proportions of outliers with >2-fold dilution differences in the susceptibility results were 1.8% for MEM, 6.4% for IPM, 2.3% for FEP, and 5.5% for SCF. For *P. aeruginosa*, the corresponding outlier rates were 7.4% (MEM), 4.2% (IPM), 8.4% (FEP), and 7.4% (SCF). These findings demonstrated that the NMIC-413 panel exhibited good accuracy in AST, with the highest precision and essential agreement observed for MEM (98.2%) susceptibility testing in *Enterobacteriaceae*.

The distribution of MICs determined by the BMD method and the inhibition zone determined by the disk diffusion method for *Enterobacteriaceae* bacteria are shown in Supplementary Figures S1A–D. For MEM and FEP, the resistance rates determined by the disk diffusion method were higher than those determined by the BMD method. For IPM and SCF, the resistance rates determined by the disk diffusion method were lower than those determined by the BMD method. The MIC distribution determined by the BMD method and the inhibition zone determined by the disk diffusion method for *P. aeruginosa* are shown in Supplementary Figures S1E–H. For MEM and FEP, the resistance rates determined by the disk diffusion method were lower than those determined by the BMD method. For IPM and SCF, the resistance rates determined by the disk diffusion method were identical to those determined by the BMD method.

## 4 Discussion

In this evaluation, the NMIC-413 panel demonstrated that the CA, EA, ME, and VME values for MEM, IPM, FEP, and SCF generally met the acceptable standards. Compared to the conventional disk diffusion method, the NMIC-413 panel exhibited significantly higher CA values for CRE/CRPA (99.3, 96.6, 98.0, and 98.7% / 98.2%, 98.2%, 96.4%, and 92.9%) than those obtained using the disk diffusion method (98.7, 96.0, 96.0, and 97.3% / 96.4, 98.2, 92.9, and 92.9%). These findings indicate that the NMIC-413 panel provides more reliable detection results overall, which is of significant clinical importance in regions with high CRE/CRPA prevalence.

In the performance evaluation of the NMIC-413 AST panel, this study demonstrated superior accuracy for MEM compared to regional studies such as Zhang et al. (2021), who reported *Enterobacteriaceae* data from North China (MEM CA 99.5% vs. 97.69%), and Tian et al. (2024), who analyzed Gram-negative bacteria from Central China (MEM CA 98.4% vs. 97.6%). These results consistently showed higher accuracy for MEM, while IPM and FEP exhibited comparable performance, potentially reflecting regional resistance pattern differences among *Enterobacteriaceae* strains in South China. Compared to the BD Phoenix NMIC-500 panel data from Korea reported by Park et al. (2019), the NMIC-413 panel demonstrated enhanced detection performance for *Enterobacteriaceae* (IPM CA 96.3% vs. 87.2%) and non-fermenters (IPM CA 97.9% vs. 92.7%), with particularly improved accuracy for IPM. Our results align with and extend previous studies conducted in China and internationally. Notably, our direct comparison of the NMIC-413 panel with both disk diffusion and BMD methods in a large clinical cohort provides new valuable validation data for routine clinical implementation.

The IDSA guidelines for treating CRE and DTR *P. aeruginosa* infections recommend the following (Tamma et al., 2022): For CRE infections, combination therapy with carbapenems could be selected based on AST, and carbapenems could be used in combination with enzyme inhibitors to delay resistance development. For DTR *P. aeruginosa*, when isolates demonstrate susceptibility to traditional non-carbapenem *β*-lactams (e.g., FEP), these agents are preferred over carbapenems. In clinical practice, Zhen et al. (2023) reported that 76% of 100 CRPA bacteremia cases (2014–2022) were still treated with conventional regimens, including carbapenems or FEP. Buyukyanbolu et al. (2025) documented that 46.5% of 244 Turkish CRPA isolates showed susceptibility to increased exposure (SIE/I) of FEP. For the CRPA isolates with an FEP MIC of 8 mg/L, optimized dosing (2 g q8h 0.5 h infusion) improved the cumulative response fraction from 86%/81 to 96%. A CFR of ≥90% generally indicated clinically acceptable efficacy.

The Chinese 2023 Guidelines for the Diagnosis and Treatment of CRGNB (Zeng et al., 2023) recommend promptly determining the MICs of commonly used antimicrobial agents for CRGNB infections in local hospitals. Empirical antimicrobial therapy should be initiated concurrently with MIC testing to ensure early intervention. The NMIC-413 panel, with its demonstrated accuracy, enables clinicians to transition from empirical broad-spectrum therapy, e.g., polymyxins (Sheu et al., 2019), to targeted treatment once MIC results are available. Notably, when the panel indicates susceptibility to carbapenems or cephalosporins, de-escalation can be safely implemented, effectively reducing unnecessary polymyxin use and the associated risk of nephrotoxicity (Mostardeiro et al., 2013; Nation et al., 2017; Xia et al., 2023). The NMIC-413 panel demonstrated exceptional reliability (Kappa> 0.9) for susceptibility testing of MEM, IPM, FEP, and SCF, supporting its clinical utility in guiding therapeutic decisions for severe infections. Its automated system minimizes subjective interpretation errors—particularly notable when compared to the disk diffusion method (e.g., improved Kappa for FEP by *Δ* = +0.065)—making it particularly valuable for multicenter studies and antimicrobial resistance surveillance networks. However, automated systems require substantial capital investment in equipment. For clinical laboratories in primary care hospitals with a low volume of samples to be processed, the disk diffusion method remains the most cost-effective option, provided strict quality control measures are implemented.

Notably, a Greek time-series study (Kousovista et al., 2021) revealed dynamic correlations between antibiotic consumption and resistance development; the use of MEM showed an immediate positive association with *P. aeruginosa* resistance (*p* < 0.001). Clinical interpretation of NMIC-413 results must consider local antibiotic usage patterns in high MEM consumption regions—e.g., Argentina’s urban areas showed significantly higher antibiotic use/resistance compared to rural zones (Boni et al., 2022). Caution is warranted even with susceptible results. Automated AST systems such as the NMIC-413 panel require integration with regional antimicrobial surveillance data to achieve truly precision medicine.

This study has several limitations that warrant consideration. Firstly, in this study, the interpretation of SCF susceptibility was based on cefoperazone breakpoints rather than internationally standardized criteria for SCF. While this approach has demonstrated validity in prior research (Barry and Jones, 1988; Jean et al., 2017; Jones et al., 1987; Jones et al., 2014), its use may affect the generalizability of our findings, and caution should be exercised when interpreting the susceptibility results. Secondly, the limited sample size of non-dominant species—including *Enterobacter cloacae* (n = 11), *Citrobacter koseri* (n = 1), and *Enterobacter hodginsii* (n = 1)—may constrain the accuracy of the performance evaluation for these organisms. Finally, insufficient consideration of regional strain variations may affect the generalizability of our findings across regions with differing resistance mechanisms. While this study supports the use of the NMIC-413 panel for AST of CRE and CRPA, continued surveillance and multicenter studies are needed before it can widely replace the gold-standard BMD methods. Future research should focus on establishing standardized SCF breakpoints, expanding rare species sampling, and conducting multicenter validation studies to assess geographical variability.

## 5 Conclusion

The NMIC-413 panel demonstrated CLSI-compliant performance for all four antibiotic classes against CRE and CRPA, showing superior reliability compared to conventional disk diffusion methods. With its proven accuracy, this panel is well-suited for implementation in clinical laboratories serving CRE/CRPA-endemic areas, enabling prompt, evidence-based antibiotic decision-making. While this study supports the use of the NMIC-413 panel for AST of CRE and CRPA, continued surveillance and multicenter studies are needed before it can widely replace the gold-standard BMD methods. Future directions should focus on standardizing SCF breakpoints, expanding rare pathogen detection, and conducting multicenter validation studies to address geographic variations—crucial steps for optimizing the NMIC-413 panel’s role in precision antimicrobial management.

## Data Availability

The original contributions presented in the study are included in the article/Supplementary material, further inquiries can be directed to the corresponding author.
