# Supplementary material for: Performance evaluation of four antibiotics using the BD Phoenix™ NMIC-413 antimicrobial susceptibility testing panel for carbapenem-resistant Enterobacteriaceae and carbapenem-resistant Pseudomonas aeruginosa
Source: Front Microbiol. 2025 Jul 4;16:1593674. doi: 10.3389/fmicb.2025.1593674 (PMC12271110; doi:10.3389/fmicb.2025.1593674)
Supplement: Supplementary file 2 [file Table_2.docx]

Supplementary Material

# Supplementary Tables and Figures

## Supplementary Tables

**Table S2** Bacterial susceptibility test performance for MEM

|  | NMIC-413 panel | | | | | |  | Disk diffusion method | | | | |
| --- | --- | --- | --- | --- | --- | --- | --- | --- | --- | --- | --- | --- |
|  | Total no. evaluable | CA (%) | EA (%) | VME (%) | ME (%) | MIE (%) |  | Total no. evaluable | CA (%) | VME (%) | ME (%) | MIE (%) |
| *Total* | 314 | 98.4 | 96.5 | 0 | 0.3 | 1.3 |  | 314 | 98.1 | 0 | 0 | 1.9 |
| *Enterobacteriaceae* | 219 | 99.5 | 98.2 | 0 | 0.5 | 0 |  | 219 | 99.1 | 0 | 0 | 0.9 |
| *CSE* ^1^ | 69 | 100 | 97.1 | 0 | 0 | 0 |  | 69 | 100 | 0 | 0 | 0 |
| *CRE* ^2^ | 150 | 99.3 | 98.7 | 0 | 0.7 | 0 |  | 150 | 98.7 | 0 | 0 | 1.3 |
| *K. pneumoniae* | 154 | 99.4 | 98.7 | 0 | 0.6 | 0 |  | 154 | 99.4 | 0 | 0 | 0.6 |
| *CSKP* ^3^ | 22 | 100 | 95.5 | 0 | 0 | 0 |  | 22 | 100 | 0 | 0 | 0 |
| *CRKP* ^4^ | 132 | 99.2 | 99.2 | 0 | 0.8 | 0 |  | 132 | 99.2 | 0 | 0 | 0.8 |
| *P. aeruginosa* | 95 | 95.8 | 92.6 | 0 | 0 | 4.2 |  | 95 | 95.8 | 0 | 0 | 4.2 |
| *CSPA* ^5^ | 39 | 92.3 | 87.2 | 0 | 0 | 7.7 |  | 39 | 94.9 | 0 | 0 | 5.1 |
| *CRPA* ^6^ | 56 | 98.2 | 96.4 | 0 | 0 | 1.8 |  | 56 | 96.4 | 0 | 0 | 3.6 |

**Table S3** Bacterial susceptibility test performance for IPM

|  | NMIC-413 panel | | | | | |  | Disk diffusion method | | | | |
| --- | --- | --- | --- | --- | --- | --- | --- | --- | --- | --- | --- | --- |
|  | Total no. evaluable | CA (%) | EA (%) | VME (%) | ME (%) | MIE (%) |  | Total no. evaluable | CA (%) | VME (%) | ME (%) | MIE (%) |
| *Total* | 313 | 96.8 | 94.2 | 0.6 | 0.6 | 1.9 |  | 313 | 95.5 | 1.0 | 0.3 | 3.2 |
| *Enterobacteriaceae* | 218 | 96.3 | 93.6 | 0.9 | 0.9 | 1.8 |  | 219 | 95.0 | 0.9 | 0.5 | 3.7 |
| *CSE* | 69 | 95.7 | 88.4 | 0 | 0 | 4.3 |  | 69 | 92.8 | 0 | 0 | 7.2 |
| *CRE* | 149 | 96.6 | 96.0 | 1.3 | 1.3 | 0.7 |  | 150 | 96.0 | 1.3 | 0.7 | 2.0 |
| *K. pneumoniae* | 153 | 96.7 | 94.8 | 1.3 | 1.3 | 0.7 |  | 154 | 96.1 | 0.6 | 0.6 | 2.6 |
| *CSKP* | 22 | 100 | 90.9 | 0 | 0 | 0 |  | 22 | 95.5 | 0 | 0 | 4.5 |
| *CRKP* | 131 | 96.2 | 95.4 | 1.5 | 1.5 | 0.8 |  | 132 | 96.2 | 0.8 | 0.8 | 2.3 |
| *P. aeruginosa* | 95 | 97.9 | 95.8 | 0 | 0 | 2.1 |  | 94 | 96.8 | 1.1 | 0 | 2.1 |
| *CSPA* | 39 | 97.4 | 92.3 | 0 | 0 | 2.6 |  | 38 | 94.7 | 0 | 0 | 5.3 |
| *CRPA* | 56 | 98.2 | 98.2 | 0 | 0 | 1.8 |  | 56 | 98.2 | 1.8 | 0 | 0 |

**Table S4** Bacterial susceptibility test performance for FEP

|  | NMIC-413 panel | | | | | |  | Disk diffusion method | | | | |
| --- | --- | --- | --- | --- | --- | --- | --- | --- | --- | --- | --- | --- |
|  | Total no. evaluable | CA (%) | EA (%) | VME (%) | ME (%) | MIE (%) |  | Total no. evaluable | CA (%) | VME (%) | ME (%) | MIE (%) |
| *Total* | 314 | 97.5 | 95.9 | 0 | 0.3 | 2.2 |  | 314 | 93.9 | 0 | 0.3 | 5.7 |
| *Enterobacteriaceae* | 219 | 97.7 | 97.7 | 0 | 0 | 2.3 |  | 219 | 94.5 | 0 | 0.5 | 5.0 |
| *CSE* | 69 | 97.1 | 98.6 | 0 | 0 | 2.9 |  | 69 | 91.3 | 0 | 1.4 | 7.2 |
| *CRE* | 150 | 98.0 | 97.3 | 0 | 0 | 2.0 |  | 150 | 96.0 | 0 | 0 | 4.0 |
| *K. pneumoniae* | 154 | 98.1 | 98.1 | 0 | 0 | 1.9 |  | 154 | 96.1 | 0 | 0 | 3.9 |
| *CSKP* | 22 | 100 | 100 | 0 | 0 | 0 |  | 22 | 95.5 | 0 | 0 | 4.5 |
| *CRKP* | 132 | 97.7 | 97.7 | 0 | 0 | 2.3 |  | 132 | 96.2 | 0 | 0 | 3.8 |
| *P. aeruginosa* | 95 | 96.8 | 91.6 | 0 | 1.1 | 2.1 |  | 95 | 92.6 | 0 | 0 | 7.4 |
| *CSPA* | 39 | 97.4 | 89.7 | 0 | 0 | 2.6 |  | 39 | 92.3 | 0 | 0 | 7.7 |
| *CRPA* | 56 | 96.4 | 92.9 | 0 | 1.8 | 1.8 |  | 56 | 92.9 | 0 | 0 | 7.1 |

**Table S5** Bacterial susceptibility test performance for SCF

|  | NMIC-413 panel | | | | | |  | Disk diffusion method | | | | |
| --- | --- | --- | --- | --- | --- | --- | --- | --- | --- | --- | --- | --- |
|  | Total no. evaluable | CA (%) | EA (%) | VME (%) | ME (%) | MIE (%) |  | Total no. evaluable | CA (%) | VME (%) | ME (%) | MIE (%) |
| *Total* | 314 | 96.8 | 93.9 | 0 | 1.6 | 1.6 |  | 314 | 95.5 | 1.0 | 1.0 | 2.5 |
| *Enterobacteriaceae* | 219 | 98.2 | 94.5 | 0 | 1.4 | 0.5 |  | 219 | 96.3 | 0.5 | 0.9 | 2.3 |
| *CSE* | 69 | 97.1 | 88.4 | 0 | 1.4 | 1.4 |  | 69 | 94.2 | 0 | 0 | 5.8 |
| *CRE* | 150 | 98.7 | 97.3 | 0 | 1.3 | 0 |  | 150 | 97.3 | 0.7 | 1.3 | 0.7 |
| *K. pneumoniae* | 154 | 99.4 | 96.8 | 0 | 0.6 | 0 |  | 154 | 96.8 | 0.6 | 0.6 | 1.9 |
| *CSKP* | 22 | 100 | 90.9 | 0 | 0 | 0 |  | 22 | 90.9 | 0 | 0 | 9.1 |
| *CRKP* | 132 | 99.2 | 97.7 | 0 | 0.8 | 0 |  | 132 | 97.7 | 0.8 | 0.8 | 0.8 |
| *P. aeruginosa* | 95 | 93.7 | 92.6 | 0 | 2.1 | 4.2 |  | 95 | 93.7 | 2.1 | 1.1 | 3.2 |
| *CSPA* | 39 | 94.9 | 89.7 | 0 | 2.6 | 2.6 |  | 39 | 94.9 | 0 | 2.6 | 2.6 |
| *CRPA* | 56 | 92.9 | 94.6 | 0 | 1.8 | 5.4 |  | 56 | 92.9 | 3.6 | 0 | 3.6 |

Note: ^1^ carbapenem-sensitive *Enterobacteriaceae (CSE)*; ^2^ carbapenem-resistant *Enterobacteriaceae* (*CRE*); ^3^ carbapenem-sensitive *Klebsiella pneumoniae (CSKP*); ^4^ carbapenem-resistant *Klebsiella pneumoniae* (*CRKP*); ^5^ carbapenem-sensitive *Pseudomonas aeruginosa* *(CSPA*); ^6^ carbapenem-resistant *Pseudomonas aeruginosa (CRPA*).

## Supplementary Figures

|  | | | |
| --- | --- | --- | --- |
| (**A**) | (**B**) | (**C**) | (**D**) |
|  | | | |
| (**E**) | (**F**) | (**G**) | (**H**) |

**Figure S1:** MICs determined by disk diffusion versus BMD methods: **A** (MEM), **B** (IPM), **C** (FEP), and **D** (SCF) show the comparison results of *Enterobacteriaceae* determined by disk diffusion and BMD methods, **E** (MEM), **F** (IPM), **G** (FEP), and **H** (SCF) show the comparison results of *P. aeruginosa* determined by disk diffusion and BMD methods. Dark green lines show the clinical breakpoints for each antibiotic.
